# Effect of light-selective sunshade net on the quality and aromatic characteristics of Cabernet Sauvignon grapes and wine: Exploratory experiment on strong solar irradiance in northwestern China

**DOI:** 10.1016/j.fochx.2022.100510

**Published:** 2022-11-14

**Authors:** Yanlun Ju, Uwamahoro Francoise, Demei Li, Yang Zhang, Bochen Liu, Miao Sun, Yulin Fang, Xiaofeng Wei

**Affiliations:** a College of Enology, Viti-viniculture Engineering Technology Center of State Forestry and Grassland Administration, Shaanxi Engineering Research Center for Viti-Viniculture, Northwest A&F University, Heyang Viti-viniculture Station, Yangling 712100, China; b College of Food Science and Engineering, Beijing University of Agriculture, Beijing 102206, China; c Domaine des Arômes, Yinchuan 750000, China

**Keywords:** Aroma, Phenolic substance, Grape, Light-selective sunshade net, Wine

## Abstract

The greenhouse effect is a global problem. In view of the intense sunlight radiation in Ningxia (an ideal wine-producing region in northwestern China), the effect of light-selective sunshade nets of different colors (black, red and white) on the quality and aromatic characteristics of grapes and wine was studied. With the treatments of different nets, the solar radiation intensity was significantly decreased. The sugar contents in both grapes and wines decreased, while the acid contents increased. The contents of total phenols, tannins and flavanols in grapes were increased, while the total flavonoids and anthocyanins were decreased. The contents of most phenolics in wine were increased. The contents of most aromas in grapes and wines under nets were higher than those in the control group. The black group usually possessed the highest variety and content. Red and black nets improved the fruity, floral and sweet aromas of grapes. The white net decreased the green and citrusy aromas.

## Highlights

• Effect of light-selective net on the quality and aromas were studied.
• Total radiation was obviously decreased.
• Sugar contents were decreased while the acid contents were increased.
• Most of the phenolic and aromatic substances contents were increased.
• Light-selective sunshade nets could probably improve the grape and wine quality.

## 1 Introduction

Ningxia is a new emerging wine production region that is attracting increasing attention. The climatic environment is believed to be perfect for producing high-quality grapes and wine. However, with increasing human activities, greenhouse effect is becoming increasingly serious, leading to rising global temperatures. Simultaneously, the destruction of the ozone layer leads to an increase in solar intensity (Menzel, Yuan, Matiu, Sparks, & Estrella, 2020), which is a global problem that also occurs in Ningxia.

Grapes is an important fruit distributed worldwide, and wine is receiving increasing attention due to its economic value, elegant taste and health benefits (Wei et al., 2020a). Phenolic compounds play an important role in the resistance of grapes to abiotic and biotic stress (Julia et al., 2019) and contribute greatly to the color, mouthfeel, quality and stability of wine (Aleixandre-Tudo, Nieuwoudt, Aleixandre, & Toit, 2018). Aroma is one of the most important sensory indicators and quality parameters for grapes and wine (Bahena-Garrido, Ohama, Suehiro, Hata, Isogai, Iwashita, Goto-Yamamoto, & Koyama, 2019). The type, content and sensory threshold of the phenolic substances and aromas affect the quality of grapes and wine to a large extent.

Grapes berries are one of the origins of the phenolic compounds and aromas in wine. The development of grapes is influenced by numerous environmental factors, such as temperature, water and hormones (Wei, Ju, Ma, Zhang, Fang, & Sun, 2020b). Thus, many cultivation measures, including water deficiency, rain shelter, hormone spraying, and fruit thinning, have been widely studied, aiming to improve the quality of grapes (Ju et al., 2019). Light is also a critical factor that influences grape quality and development. Light affects the photosynthesis, optimal development, sugar accumulation, berry ripening and other metabolic processes of grapevines (Sun et al., 2019). Cultivation measures, such as canopy pruning, leaf removal and cluster bagging, which can change the light conditions, have also been studied by several authors (Alem, Rigou, Schneider, Ojeda, & Torregrosa, 2018). However, in some regions, such as Ningxia, the light intensity may be too high so that it would damage the grape berry, thereby influencing the quality of grapes and wines. Similarly, too high of a temperature also has the same influence (Greer, Rogiers, & Mudiyanselage, 2019).

A light-selective sunshade net is one of the cultivation measures that can change the light quality in vineyards. This cultivation measure has been studied by Shahak (2014) in many crops in Israel, such as kiwifruit, banana, sweet pepper, apple, pear, and persimmon, for approximately 20 years. In addition to their insect-proof and hail-proof functions, it was suggested that light-selective sunshade nets can alter light conditions and simultaneously change the temperature and humidity. Consequently, these nets influence the quality of crop fruits in many ways. This influence has attracted the attention of many other researchers Milczarek, Avena-Mascareno, Jérôme, and Mélissa (2016). Basile, Giaccone, Shahak, Forlani, and Cirillo (2014) studied the comprehensive influences of light-selective sunshade nets of different colors on the development of kiwifruits. Compared to the nets of other colors, they found that the red nets stimulated vegetative growth and played a positive role on fruit size and dry matter of kiwifruits. Thus, they suggested that red nets were the most cost-effective. However, knowledge of the effect of light-selective sunshade nets on the quality of grapes and wine is limited, especially in Ningxia. Thus, in this study, we take Cabernet Sauvignon grape, which is a representative variety widely-grown in Ningxia, as the materials. Grapevines were grown under red, white and black light-selective sunshade nets, and the phenolic substances and aromas in grapes and wines were analyzed. The aim of this study was to provide a further understanding of the influence of light on grapes and wine and to study the feasibility of improving grape and wine quality by using light-selective sunshade nets.

## 2 Materials and methods

### 2.1 Plant materials

The Lilan Winery (105°58′38″E, 38°16′42″N, 1150 m above sea level), located in Yong-Ning County, the Ningxia Hui Autonomous Region, was used as the experimental site. Grapevines were planted in 2013 and grown on a north-south line with a spacing of 0.8 m × 3.0 m. The short pruning (double guyot) combined with vertical shoot positioning was used as the trellis system. The trickle irrigation system was adopted in the irrigation regimes. The vineyard soil contained about 80 % sandy soil and 15 % gravel.

Ten lines of grapevines were shaded by red (R), white (W) and black (B) light-selective sunshade nets, as shown in Fig. S1. The width of the nets was 1.3 m. Shading treatment was applied 2 weeks after flowering and 60 grapevines were randomly selected for each treatment. All the fruit parts and 90 % of the leaf curtain layer both in the east and west was effectively covered by the nets. Grapes under direct sunlight exposure were the control (CK). Grape materials were collected randomly from the beginning of véraison for two consecutive years (2016 and 2017). Sampling was conducted once every 2 weeks.

### 2.2 Winemaking process

The winemaking process was performed according to the procedure described in our previous study (Sun et al., 2018). The harvesting time was determined in terms of the average total soluble solids reaching 24 °Brix. The harvest dates between two years were not same, but similar (about September 25). The grapes were destemmed and crushed immediately after harvest. The must was poured into a 10 L glass pot with sulfur dioxide (20 mg/L) and yeast (LALVIN, Denmark, commercial strain, 0.25 g/L) added. The whole fermentation process was conducted in an environmental chamber with temperature controlled at about 20–25 °C. The wine was stored at 4 °C until analysis.

### 2.3 Chemicals and apparatus

Pure standards were purchased from Sigma-Aldrich Chemical Co. (Shanghai, China). Solvents of spectrophotometric grade were obtained from J & K Co. Ltd. (Beijing, China). A DB-Wax gas chromatography column was purchased from J & W Scientific, Rancho Cordova, CA. All the other chemicals were of analytical grade.

### 2.4 Determination of basic physicochemical parameters

The intensity of UV-A and UV-B radiation was detected by a UV detector. The intensity of total radiation was detected by a lux meter. Diameters were detected using a Vernier caliper. The Brix value was measured using a hand refractometer. The total acidity and total sugar and alcohol contents were quantified according to OIV (2012). The pH was measured with a Mettler Toledo FE20 desktop pH meter (Mettler Toledo Instruments Co. Ltd., Shanghai, China).

### 2.5 Phenolic content determination

Phenolic components in grape skins, seeds and wines were detected. The total polyphenol (TP) content was determined using the Folin-Ciocalteu method and expressed as mg gallic acid/g dry weight (DW). The total flavonoid (TFD) content was detected by the Rutin method and expressed as mg (+)-catechin/g DW (Meng, Fang, Qin, Zhuang, & Zhang, 2012). The total flavanol (TFA) content was determined with p-DMACA and expressed as mg (+)-catechin/g DW. The total anthocyanin content (TA) was estimated using the pH differential method and expressed as mg (+)-catechin/g DW (Meng et al., 2012). Total tannin (TT) was determined according to the bovine serum albumin precipitation method and expressed as mg (+)-catechin/g DW (Harbertson, Picciotto, & Adams, 2003).

### 2.6 Extraction and GC–MS analysis of aromas

Aroma extraction and detection were carried out as previously described (Ju, Liu, Zhao, Meng, & Fang, 2016). One hundred grape berries were blended with 1 g of PVPP in liquid nitrogen. Samples were macerated for 2.5 h at 4 °C and then centrifuged at 10000 rpm and 4 °C for 4 min. Then, 1 g of NaCl was added to 5 mL of clear juice along with 20 μL of the 2-octanol internal standard and blended in a 15 mL sample vial for further determination.

For solid-phase microextraction (SPME), the extract was heated at 250 °C for 2 h. The aromas were extracted in a 40 °C water bath for 30 min and subsequently desorbed at 230 °C for 3 min into the splitless injection port of a GC–MS instrument fitted with an HP-INNW AX column (0.25 mm I.D., 60 m, 0.25 m; Agilent, Shanghai, China).

Compound profiling was performed using an Agilent 6890 GC–MS system equipped with an Agilent 5975 mass spectrometer and an HP-INNOWAX capillary column (60 mm × 0.25 mm) of 0.25-mm film thickness as described previously (Wei, Ma, Cao, Sun, & Fang, 2018). The flow rate of the carrier gas (helium) was 1 mL/min. The temperature was increased from 40 °C (3 min hold) to 160 °C at a speed of 4 °C/min, then increased from 160 °C to 230 °C at 7 °C/min, and held at 230 °C for 8 min. The operating conditions are described as follows: capillary direct interface temperature, 230 °C; heater valve temperature, 245 °C; transfer line temperature, 255 °C. The trap temperature was set at −30 °C for the starting temperature and then increased to 255 °C at 40 °C/min. Samples were analyzed in triplicate.

### 2.7 Statistical analysis

Data are reported as the mean ± standard deviation (SD) values, and the statistical analysis of the data was performed using the IBM SPSS Statistics 21.0 package for Windows (SPSS, Chicago, USA). One-way analysis of variance (ANOVA) and Duncan’s multiple range tests were used to determine the significance of the difference among samples, with a significance level of 0.05, by considering the contents of different substances in grapes and wine as dependent variables and the black net, red net, white net and CK treatments as categorical factors.

## 3 Results and discussion

### 3.1 Solar radiation intensities

The intensities of UV-A, UV-B and total radiation are shown in Fig. S2. The results obtained for two years show that the light-selective sunshade net could significantly decrease the intensity of UV-A and UV-B radiation, while the white net decreased the intensity of UV-A and UV-B radiation the most. The total radiation intensity also obviously decreased, while the decrease in total radiation intensity achieved by the black net was the most significant. This result was consistent with the studies by Lobos et al. (2013), indicating that light-selective sunshade nets are possibly ideal materials for decreasing the intensity of solar radiation.

### 3.2 Basic physiological indexes of grapes and wines

As shown in Table S1, the transverse and vertical diameters of grapes grown in 2016 under red and white light-selective sunshade nets were significantly higher than those of the control group, while no significant difference was observed between the diameters of the black and control groups. However, in 2017, the diameters of grapes grown under red, white and black nets were all significantly higher than the grape diameter of the control. The grapes grown under black nets had the largest diameters. However, in general, the influence was not very large. This result is similar to the results of Shahak et al. (2009), indicating that the light-selective sunshade net treatment was possibly conducive to the growth and development of grape berries. In terms of chemical indicators, Goren et al. (2010) found that the sugar content in sweet bell pepper was not affected by colored shade nets. However, similar to the study by Basile et al. (2012), we found that the Brix value of grapes grown under all sunshade nets of different colors was significantly lower than that of the control group, while the total acidity values of grapes grown under all the sunshade nets were dramatically higher than the total acidity of the control group. Accordingly, all the pH values of grapes grown under sunshade nets were markedly lower than the pH of the control group (Table S1). The results of the wines were similar (Table S1). These interesting results indicated that treatment with light-selective sunshade nets, especially black nets, potentially contributed to decreasing the sugar content in grape berries and wines and increasing the acid content. Studies of several other researchers suggested that the wine grape in Ningxia usually faced with this problem of relative high sugar and low acid (Ju et al., 2019, Li et al., 2011, Liu et al., 2006). Thus, this treatment method is a potentially great way to solve the quality problem of grapes with a relatively high content of sugar and low acid content during harvest, which is thought to be detrimental to the quality of wine in the wine-producing regions of northwestern China, such as Ningxia and Xinjiang. Solomakhin and Blanke (2010) also found similar results in apples, indicating that the light-selective sunshade net is expected to be an excellent cultivation management measure in vineyards and helpful for obtaining grapes and wines with ideal high quality.

### 3.3 Phenolic compounds

#### 3.3.1 Phenolic compounds in grapes

**Fig. 1:**
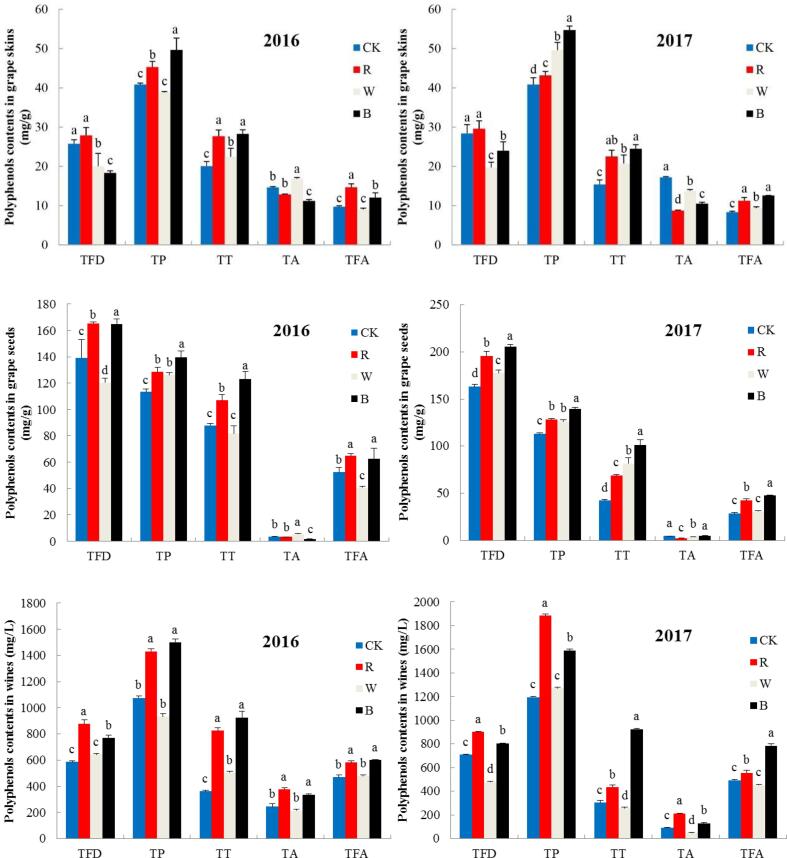
Polyphenols contents in grape skins, seeds and wines under different treatments. R, red. W, white. B, black. CK, control group. TFD, total flavonoids. TP, total phenols. TT, total tannins. TA, total anthocyanins. TFA, total flavanols. Different letters indicate significantly difference values (p < 0.05). (For interpretation of the references to color in this figure legend, the reader is referred to the web version of this article.)

Phenolic compounds in grapes were proven to be influenced by light conditions (Sun et al., 2019). As shown in Fig. 1, the content of TFD in the skins of grapes grown under the red net was the highest in 2016, followed by that of the CK group, the white net group and, finally, the black net group. In 2017, the TFD content in the grapes grown under black nets was higher than that of grapes grown under white nets. Thus, the red net could probably improve the TFD content in grape skins, while the white and black nets were counterproductive. The TP content was the highest in the skins of grapes grown under the black net in 2016, followed by that of the red net-treated group, while no significant difference was observed between the white net-treated and CK groups. In 2017, the TP contents of all three net groups were obviously higher than the TP content of the CK group, while the black net-treated group had the highest content (Fig. 1). This result is different from the study conducted by Basile et al. (2012) in kiwifruit. The trends of TT contents were similar in 2016 and 2017. The TT contents in the grape skins of all three net-treated groups were significantly higher than the TT content of the CK group, while the black net- and red net-treated groups shared the highest contents (Fig. 1). The highest content of TA in 2016 was observed in the white net-treated group, followed by the CK, red net-treated and black net-treated groups. However, in 2017, CK had the highest TA content, followed by the white net-, black net-, and red net-treated groups. This result is consistent with the study by Guan et al. (2016), who suggested that light exclusion reduces the concentration of TA in grapes. Ma, Li, Mao, Li, Zuo, Zhao, Dawuda, and Shi (2019) found the same results. For TFA, the content of red net-treated group was the highest, followed by the black net-treat group, while no obvious difference was observed between the CK and white net-treated groups. In 2017, the TFA contents of the three net-treated groups were all significantly higher than the TFA content of the CK group, while the black net-treated group possessed the highest content (Fig. 1). In summary, the light-selective sunshade net treatment, especially the black net treatment, could improve the contents of TP, TT and TFA in grape skins, while the contents of the majority of TA decreased with light-selective sunshade net treatment. The TFD content of grape skins could be improved by the red sunshade net, but the difference from that of the control group was not significant. However, previous studies have found that sunlight can promote the accumulation of flavonols in grape peel (Allegro, Pastore, Valentini, & Filippetti, 2019), while shading of grape clusters can lead to the reduction of flavonols content (Sun et al., 2019). Anić, Osrečak, Andabaka, Tomaz, Večenaj, Jelić, Kozina, Kontić, and Karoglan (2021) also found that leaf removing can significantly increase the concentration of phenols, anthocyanins, flavonols and flavanols in grape fruits. These are different from the results of our study. The reason may be that the shading net treatment in this paper not only changed the light, but also changed the temperature, humidity and other ecological factors (Kovalenko, Tindjau, Madilao, & Castellarin, 2021).

The highest content of TFD in grape seeds from 2016 was achieved by the black net-treated group, followed by the red net-treated, CK and white net-treated groups. In 2017, the TFD contents of the three net-treated groups were all significantly higher than the TFD content of the CK group. This finding was different from the trend observed for the skins (Fig. 1). Similar TP content trends were observed in 2016 and 2017 in grape seeds. The TP contents of all three net-treated groups were significantly higher than the TP content of the CK group, while the black net-treated group had the highest TP content. The TT content of the black net-treated group was the highest in 2016, followed by that of the red net-treated group, while no obvious difference was observed between the CK and white net-treated groups. However, in 2017, the TT contents of all three net-treated groups were higher than the TT content of the CK group, with an order of black net-treated, white net-treated, red net-treated and CK (Fig. 1). The TFA contents of all three net-treated groups were significantly higher than the TFA content of the CK group in 2016, while no obvious difference was observed between the TFA contents of the red net-treated and black net-treated groups. In 2017, the black net-treated groups possessed the highest TFA contents, while no significant difference was found between the white net-treated and CK groups. Generally, light-selective sunshade nets, especially black and red nets, could improve the contents of TFD, TP, TT and TFA in grape seeds. The active influence of white nets was not very obvious. However, Savikin et al. (2013) found that the contents of TP, TFD and TT in black currant were relatively lower than those in the control, which is slightly different from our results. This result may be because of the differences in species and climatic conditions in different production areas.

#### 3.3.2 Phenolic compounds in wines

Phenolic substances contribute greatly to wine quality (Tian, Harrison, Morton, & Jaspers, 2020). In 2016, the TT contents in wines produced from grapes grown under the three different nets were significantly higher than the TT content of the CK group, while no large difference was observed between the red net-treated and black net-treated groups (Fig. 1). However, in 2017, the trend was slightly different. As shown in Fig. 1, the black net-treated group had the highest content of TT, followed by the red net-treated, CK and white net-treated groups. Moreover, the TT content of the black net-treated group was 2–3 times higher than that of the other three groups. There was no significant difference between the TP contents of the black net-treated and red net-treated groups in 2016, while the same phenomenon was also observed for the CK and white net-treated groups. In 2017, the red net-treated group possessed the highest TP content, followed by the black net-treated group, while no obvious difference in TP content was observed between the CK and white net-treated groups (Fig. 1). In both 2016 and 2017, the red net-treated group had the highest content of TFD, followed by the black net-treated group. No substantial difference in TFD content was found between the CK and white net-treated groups in 2016, while the TFD content of the CK group was significantly higher than that of the white net-treated group in 2017 (Fig. 1). Similarly, no obvious difference in TA content was observed between the CK and white net-treated groups, while the red net-treated group had the highest content in 2016. In 2017, the highest content of TA was also observed for the red net-treated groups, followed by the black net-treated, CK and white net-treated groups (Fig. 1). Overall, for the two years examined, the red and black nets were believed to be beneficial to the accumulation of TT, TP, TFD and TA in wine, while the promotion effect of the black net on TT content might be very significant.

### 3.4 Aroma components

#### 3.4.1 Aroma components in grapes

The effect of light-selective sunshade nets on aroma contents has been studied in different species, such as tomatoes (Tinyane, Sivakumar, & Soundy, 2013) and sweet peppers (Selahle, Sivakumar, Jifon, & Soundy, 2015). However, similar studies on grapes are scarce. Table 1 shows the content of volatile components in grapes. The detected aroma compounds were mainly divided into four groups: esters, alcohols, acids and others. There were 17 kinds of volatile compounds, including 4 esters, 8 alcohols, 3 acids and 2 others.

**Table 1:** Content of volatile components in grapes (μg/L).

Compounds	2016				2017			
	R	W	B	CK	R	W	B	CK
*Esters*								
Ethyl Acetate	9.33 ± 1.04a	6.45 ± 0.14c	7.81 ± 0.62b	4.04 ± 0.07d	12.48 ± 1.27c	17.15 ± 1.01b	23.20 ± 2.68a	10.05 ± 0.07c
Isoamyl acetate	18.06 ± 3.09b	18.89 ± 1.44b	22.47 ± 1.85a	15.16 ± 2.30c	34.71 ± 6.36a	24.82 ± 2.88b	38.66 ± 4.01a	25.77 ± 2.02b
Ethyl octanoate	17.76 ± 3.96b	18.80 ± 1.73ab	20.64 ± 1.11a	16.55 ± 1.52b	24.37 ± 4.25b	27.16 ± 0.87b	35.01 ± 1.94a	37.34 ± 5.53a
Heptanoic acid, 2-ethyl-	0.19 ± 0.01b	ND	0.31 ± 0.01a	ND	0.52 ± 0.01	ND	ND	ND
Total Esters	45.34 ± 5.06b	44.14 ± 3.96b	51.23 ± 1.51a	35.75 ± 2.94c	72.08 ± 11.05b	69.13 ± 3.36b	96.87 ± 3.90a	73.16 ± 6.87b

*Alcohols*								
Ethanol	86.49 ± 6.54a	73.23 ± 2.17c	85.94 ± 7.47a	79.36 ± 5.22b	96.16 ± 13.39c	64.85 ± 1.71b	66.14 ± 5.29b	106.00 ± 12.73a
Isobutyl alcohol	2.42 ± 0.08b	6.34 ± 1.15a	6.87 ± 1.37a	1.57 ± 0.01c	8.71 ± 1.01b	2.04 ± 0.04c	11.37 ± 1.30a	ND
1-Hexanol	27.31 ± 5.49d	38.20 ± 3.02b	45.39 ± 4.48a	31.77 ± 2.77c	12.76 ± 1.03c	24.05 ± 0.85b	39.82 ± 3.44a	32.85 ± 2.08a
Isooctanol	ND	47.53 ± 4.39a	44.34 ± 1.07a	ND	ND	24.04 ± 5.21b	68.28 ± 1.71a	11.77 ± 0.48c
1-Octanol	1.12 ± 0.02b	1.31 ± 0.01b	2.06 ± 0.64a	0.78 ± 0.41c	1.03 ± 0.01b	0.52 ± 0.01c	1.75 ± 0.02a	ND
(E)-2-Hexen-1-ol	25.36 ± 2.10c	24.98 ± 2.55c	40.02 ± 4.84a	31.11 ± 1.68b	30.12 ± 2.87c	38.66 ± 0.86b	51.13 ± 7.18a	40.42 ± 4.34b
Benzyl Alcohol	ND	0.07 ± 0.01	ND	ND	ND	0.19 ± 0.01b	1.31 ± 0.04a	ND
Phenylethyl Alcohol	2.54 ± 0.69b	3.19 ± 0.14b	6.81 ± 1.20a	2.25 ± 1.11b	3.84 ± 0.98a	1.28 ± 0.01b	4.53 ± 0.24a	ND
Total Alcohols	145.24 ± 5.52c	194.85 ± 12.07b	231.43 ± 3.89a	146.84 ± 6.76c	152.62 ± 2.79c	155.63 ± 13.10c	244.33 ± 9.47a	191.04 ± 18.81b

*Acids*								
Acetic acid	12.05 ± 1.27c	14.88 ± 1.06b	17.73 ± 2.53a	7.70 ± 0.23d	13.75 ± 0.95c	18.99 ± 1.07b	26.36 ± 5.00a	15.50 ± 2.33b
Hexanoic acid	3.48 ± 0.88c	ND	15.63 ± 4.01a	3.19 ± 1.57b	0.89 ± 0.01c	2.33 ± 0.02b	12.71 ± 1.08a	ND
Octanoic Acid	2.24 ± 0.04b	2.07 ± 0.63b	3.69 ± 1.31a	2.01 ± 0.10ab	2.59 ± 0.54b	1.41 ± 0.26c	5.35 ± 1.05a	1.07 ± 0.01d
Total Acids	17.77 ± 1.07b	16.95 ± 0.85b	37.05 ± 3.86a	12.90 ± 1.17b	17.23 ± 1.08c	22.73 ± 0.84b	44.42 ± 3.32a	16.57 ± 1.58bc

*Others*								
2-methyl-Cyclopentanone	214.36 ± 13.84b	225.12 ± 10.66b	525.66 ± 46.57a	45.37 ± 8.25c	123.95 ± 5.45b	ND	842.10 ± 46.69a	ND
Hexanal	102.75 ± 9.93a	34.69 ± 4.58c	84.21 ± 4.04b	77.12 ± 5.75b	44.35 ± 4.60c	ND	35.14 ± 2.79a	53.41 ± 5.21b
Total Others	317.11 ± 21.08b	259.81 ± 13.87b	609.87 ± 32.45a	122.49 ± 10.50c	168.30 ± 8.42b	ND	949.23 ± 63.57a	53.41 ± 5.21c
Total	525.46 ± 14.68b	515.75 ± 37.82b	929.58 ± 42.87a	319.98 ± 20.54c	410.23 ± 16.33b	247.49 ± 14.16d	1262.85 ± 63.24a	331.18 ± 21.05c

Note: Mean values (SD, *n* = 3) of the same compounds followed by different letters are significantly different (p < 0.05). ND, not detected. R, red. W, white. B, black. CK, control. Different letters indicate significantly difference values (p < 0.05).

In 2016, except for ethyl octanoate, the contents of most esters in grapes grown under different nets were higher than those of the CK group. The contents of total esters in grapes grown under sunshade nets were all higher than those of the CK group. In 2017, the contents of almost all kinds of esters, including the total esters, in grapes grown under the black net were higher than those of the other 3 groups. There were generally more esters in grapes grown under red and black nets than in the other two groups. Ji et al. (2019) found that colored paper bags could promote the accumulation of esters but decrease the contents of other aromatic substances. The relative mechanism needs future study. The contents of all alcohols except ethanol, 1-hexanol and (E)-2-hexen-1-ol in the grapes grown under different kinds of sunshade nets were significantly higher than those of the CK group (Table 1). Grapes grown under black nets had the highest contents of all alcohols except ethanol and isooctanol. Generally, there were more alcohols in the grapes grown under black and white nets than in the other 2 groups.

In 2016, the contents of almost all kinds of acids in grapes grown under different nets were markedly higher than those of the control group (Table 1). The total acidity of grapes grown under both the red and white nets was higher than that of the CK group, but there was no significant difference between these 2 groups. The total acidity of grapes grown under black nets was significantly higher than that of the other 3 groups. This result is consistent with the results of the basic indexes (Table S1). A similar trend was found in grapes in 2017 (Table 1). Acid contents in grapes grown under black and white nets were observably higher than the acid content of the CK group, while the grapes grown under black nets had the highest content of acids. No significant difference was observed between the red net-treated and CK groups.

No other volatile substance was detected in the grapes grown under a white net in 2017. In addition, the total contents of the other substances in grapes grown under different sunshade nets were all significantly higher than those of the CK group. In both 2016 and 2017, grapes grown under black sunshade nets had the highest content of other aromas (Table 1). The total contents in grapes grown under all kinds of nets were markedly higher than those of the CK group in 2016, while grapes grown under black nets had the highest content. In contrast, in 2017, the total content of aromas in grapes grown under black nets was the highest, followed by the red net-treated, CK and white net-treated groups (Table 1).

Aroma characteristics of grapes. (A), PCA analysis for the effect of vintage on aroma substances. (B), PCA analysis for the effect of treatment on aroma substances. (C) RDA/CCA analysis for the characteristic aroma substances. (D) Random forest analysis for the characteristic aroma substances.

**Fig. 2:**
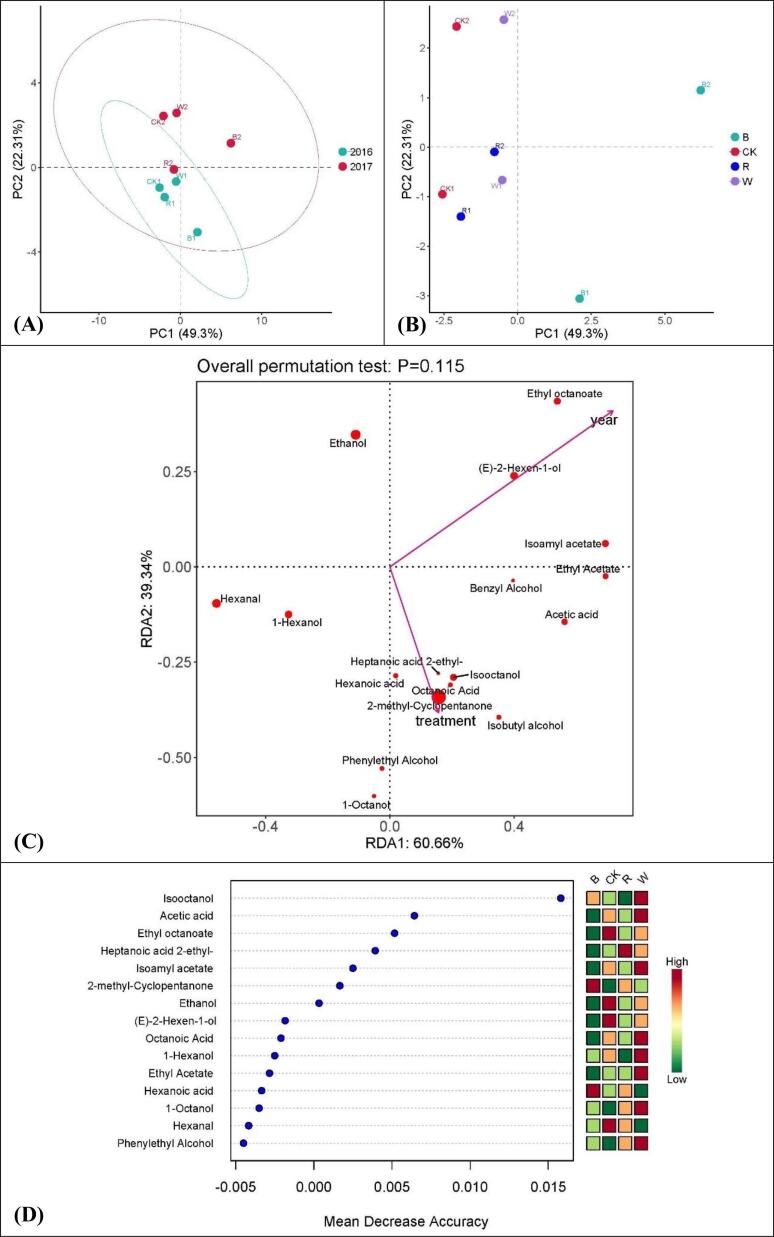
Aroma characteristics of grapes. (A), PCA analysis for the effect of vintage on aroma substances. (B), PCA analysis for the effect of treatment on aroma substances. (C), RDA/CCA analysis for the characteristic aroma substances. (D), Random forest analysis for the characteristic aroma substances. R, red. W, white. B, black. CK, control group. (For interpretation of the references to color in this figure legend, the reader is referred to the web version of this article.)

In PCA analysis, PC1 and PC2 explained 49.30 % and 22.31 % of the total variance, respectively (Fig. 2A and B). In terms of vintage, grape samples were clearly distinguished by PC2, while PC1 showed low discrimination to samples (Fig. 2A). As far as treatment is concerned, B were distinctly distinguished from the other groups by PC1, while samples of different vintages were clearly distinguished by PC2 (Fig. 2B). According to the RDA/CCA analysis results, high degree of correlation was observed for aroma substances contents with both vintage and treatment methods. It was also found that there is a negative correlation between the vintage and treatment method. Ethyl octanoate, (E)-2-Hexen-ol, Isoamyl acetate, Ethyl acetate, Benzyl alcohol, Hexanal and 1-Hexanol were influenced more by the vintage, while the other substances were more affected by the treatment methods. The contents of Ethanol, Hexanal, (E)-2-Hexen-ol, 1-Hexanol, Isooctanol, 2-methyl-Cyclopentanone, Acetic acid, Ethyl octanoate, Ethyl acetate and Isoamyl acetate apparently higher than that of the other substances (Fig. 2C). The contribution of Isooctanol to the grape aromas was significantly higher than that of other substances, followed by Acetic acid, Ethyl octanoate, Heptanoic acid 2-ethyl-, Isoamyl acetate, 2-methyl-Cyclopentanone and Ethanol. Other substances contributed relatively small to the grape aromas (Fig. 2D).

In general, the contents of most of the aromas in grapes grown under light-selective sunshade nets were higher than those of the CK group. Grapes grown under nets also have greater varieties of aromas. The black net-treated group usually had the greatest variety and highest content of aromas. This finding is consistent with the studies by Selahle et al. (2015). Zhang et al. (2017) also found that supplementary light was beneficial for the accumulation of volatile aldehydes and ketones. All these results indicated that light-selective sunshade nets are possibly an ideal cultivation measure to improve the varieties and contents of aromatic substances.

#### 3.4.2 Aroma components in wines

Aromas are important to wine quality (Castilhos, Bianchi, Gómez-Alonso, García-Romero, & Hermosín-Gutiérrez, 2020). Few studies have been performed to study the influence of light-selective sunshade nets on the aromas in wine. Table 2 shows the content of volatile components in wines. Thirty-eight kinds of aromas, including 13 esters, 11 alcohols, 9 acids and 5 others, were detected.

**Table 2:** Content of volatile components in wines (μg/L).

Compounds	2016				2017			
	R	W	B	CK	R	W	B	CK
*Esters*								
Ethyl acetate	102.64 ± 8.60a	85.41 ± 2.44b	93.76 ± 7.31ab	55.87 ± 8.98c	154.06 ± 14.30b	188.95 ± 2.36a	174.54 ± 11.24a	96.90 ± 7.61c
Ethyl butyrate	8.44 ± 1.32b	20.04 ± 2.10a	21.78 ± 3.28a	9.36 ± 1.11b	13.62 ± 1.02b	9.49 ± 0.27c	17.51 ± 3.62a	10.33 ± 1.15c
Isoamyl acetate	353.96 ± 12.46c	328.04 ± 8.22bc	469.93 ± 10.84a	345.58 ± 3.78b	658.98 ± 29.74a	436.39 ± 4.93c	509.12 ± 11.52b	324.19 ± 16.55d
Phenacyl thiocyanate	12.07 ± 1.08b	ND	15.88 ± 1.13a	ND	ND	ND	21.94 ± 8.82	ND
Ethyl octanoate	328.20 ± 7.99c	303.14 ± 10.54c	421.87 ± 15.06a	371.09 ± 23.21b	386.98 ± 5.09b	262.05 ± 1.33c	491.47 ± 17.54a	450.35 ± 22.62a
Hexanedioic acid, dioctyl ester	1.73 ± 0.02a	0.18 ± 0.01b	2.02 ± 0.01a	ND	ND	ND	3.19 ± 0.31	ND
Hexyl acetate	1.11 ± 0.03b	ND	2.64 ± 0.15a	0.48 ± 0.01c	1.30 ± 0.17	ND	ND	ND
Ethyl hexanoate	13.26 ± 3.81c	20.33 ± 2.24b	21.47 ± 4.07a	20.86 ± 3.33b	22.32 ± 0.56a	17.22 ± 2.64b	16.07 ± 2.24b	17.55 ± 1.75b
Geranyl acetate	7.76 ± 1.38b	ND	9.88 ± 0.75a	5.31 ± 1.22c	5.35 ± 0.07a	2.09 ± 0.14b	5.61 ± 1.23a	ND
Ethyl butanoate	1.32 ± 0.01a	0.75 ± 0.01bc	0.89 ± 0.01b	0.66 ± 0.05c	1.26 ± 0.21b	1.17 ± 0.06b	1.78 ± 0.11a	0.63 ± 0.01c
Ethyl heptanoate	6.19 ± 1.62b	ND	13.67 ± 1.55a	ND	4.81 ± 0.81	ND	ND	ND
Ethyl decanoate	35.74 ± 5.31d	188.66 ± 25.64b	113.07 ± 11.04c	203.21 ± 4.05a	77.64 ± 1.18bc	124.43 ± 12.54a	65.80 ± 4.49c	84.27 ± 5.07b
2-Methylbutyl acetate	4.88 ± 0.46c	3.67 ± 1.11d	6.20 ± 0.58a	5.35 ± 1.17b	5.50 ± 0.37a	4.19 ± 1.04c	4.85 ± 0.04b	4.33 ± 0.53c
Total Esters	881.16 ± 44.41b	955.47 ± 8.19c	1198.037 ± 82.52a	1017.77 ± 11.48b	1333.10 ± 42.64a	1046.01 ± 20.34b	1313.88 ± 18.10a	989.72 ± 13.68b

*Alcohols*								
Ethanol	2437.40 ± 72.69a	1558.75 ± 55.17c	2769.64 ± 34.16a	1836.29 ± 15.22b	1697.81 ± 31.66b	5797.73 ± 12.25a	6450.05 ± 46.37a	1643.09 ± 7.64b
Isobutyl alcohol	20.92 ± 2.72b	52.39 ± 10.39a	58.11 ± 13.87a	9.74 ± 1.20c	43.54 ± 2.62b	77.97 ± 4.01a	69.40 ± 7.68a	31.97 ± 1.03c
3-methyl-1-Pentanol	ND	1.30 ± 0.01b	2.42 ± 0.02a	0.88 ± 0.01c	0.69 ± 0.02b	2.63 ± 0.34a	ND	ND
3-(Methylthio)-1-propanol	0.27 ± 0.04c	1.14 ± 0.06a	1.37 ± 0.15a	0.61 ± 0.01b	1.24 ± 0.04a	0.92 ± 0.10b	0.84 ± 0.05b	ND
Isoamyl alcohol	1039. 22 ± 78.76c	1297.63 ± 54.25b	1834.44 ± 22.24a	928.17 ± 21.76c	1815.36 ± 37.28a	517.78 ± 10.17c	1749.16 ± 20.77a	1064.11 ± 13.63b
1-Hexanol	41.71 ± 4.62b	43.36 ± 7.33b	50.16 ± 2.58a	41.99 ± 2.41b	48.25 ± 4.58b	41.24 ± 1.32c	66.90 ± 6.93a	41.93 ± 1.01c
Isooctanol	ND	13.22 ± 2.08b	18.29 ± 4.83a	6.68 ± 1.16c	3.28 ± 0.11b	0.96 ± 0.06c	8.46 ± 1.64a	2.38 ± 0.54b
2,3-Butanediol	17.17 ± 2.94b	15.33 ± 4.65c	19.54 ± 1.20a	14.92 ± 3.33c	24.21 ± 3.67a	21.51 ± 1.56a	21.30 ± 0.81a	10.12 ± 1.06b
1-Octanol	5.92 ± 0.87bc	6.60 ± 0.44b	8.11 ± 2.72a	4.54 ± 0.49c	6.34 ± 1.28a	4.81 ± 1.10b	6.13 ± 0.55a	3.94 ± 0.62b
1-Undecanol	2.83 ± 0.42bc	3.36 ± 0.55b	5.65 ± 1.47a	2.77 ± 0.30c	1.44 ± 0.19b	0.57 ± 0.08c	1.31 ± 0.20b	6.15 ± 0.46a
Phenylethyl alcohol	362.07 ± 6.07c	467.47 ± 18.66b	558.15 ± 33.37a	333.92 ± 25.86c	393.61 ± 10.33a	8.89 ± 0.59b	325.32 ± 7.98a	ND
Total Alcohols	3927.51 ± 37.22b	3460.55 ± 84.41bc	5325.88 ± 103.27a	3180.51 ± 66.70c	4035.77 ± 21.74c	6475.01 ± 32.85b	8698.87 ± 83.34a	2803.69 ± 30.48d

*Acids*								
Succinic acid	113.94 ± 25.63b	97.24 ± 8.82c	152.02 ± 13.67a	88.61 ± 5.11d	308.98 ± 13.53a	90.53 ± 4.52c	222.07 ± 8.66b	13.16 ± 1.20d
Acetic acid	30.03 ± 3.39b	31.82 ± 1.45b	36.49 ± 8.46a	22.54 ± 2.82c	41.86 ± 4.19b	37.30 ± 1.86c	48.90 ± 8.36a	12.58 ± 0.41c
Isobutyric acid	0.37 ± 0.01c	0.69 ± 0.02b	0.85 ± 0.01a	0.33 ± 0.01c	1.41 ± 0.06b	1.88 ± 0.11a	1.61 ± 0.09ab	ND
3-methyl-Butanoic acid	4.40 ± 1.20a	ND	4.39 ± 0.58a	3.51 ± 0.43b	6.93 ± 1.17a	5.59 ± 0.53b	4.55 ± 0.25c	ND
2-Quinolinecarboxylic acid	3.57 ± 0.48b	3.14 ± 0.27b	5.85 ± 1.11a	ND	ND	6.15 ± 0.24a	4.57 ± 0.08b	3.93 ± 0.51c
Hexanoic acid	11.19 ± 2.73c	15.97 ± 3.87bc	34.61 ± 7.18a	18.74 ± 1.04b	23.65 ± 1.33a	18.04 ± 0.27b	21.72 ± 0.05a	13.13 ± 0.79c
Octanoic acid	30.74 ± 4.95b	29.38 ± 2.55b	45.52 ± 7.08a	25.13 ± 2.22c	56.08 ± 2.99b	48.46 ± 0.16c	63.07 ± 4.64a	30.79 ± 5.42d
Undecylenic acid	2.00 ± 0.50a	1.77 ± 0.44b	2.62 ± 0.07a	0.89 ± 0.01c	1.92 ± 0.10a	ND	ND	0.62 ± 0.03b
11-Bromoundecanoic acid	0.11 ± 0.01b	ND	1.35 ± 0.05a	0.16 ± 0.01b	1.02 ± 0.02	ND	ND	ND
Total Acids	196.35 ± 24.31b	180.01 ± 15.42b	283.70 ± 38.33a	159.91 ± 17.71c	441.85 ± 23.37a	207.95 ± 15.74c	366.49 ± 10.69b	74.21 ± 8.25d

*Others*								
Styrene	22.47 ± 4.42b	24.13 ± 2.45b	32.28 ± 5.19a	19.70 ± 4.67c	27.51 ± 0.14a	22.43 ± 2.33b	12.67 ± 0.76c	28.17 ± 1.02a
*β*-cyclocitral	31.01 ± 3.76a	11.20 ± 1.08c	26.82 ± 2.27a	16.45 ± 3.36b	258.85 ± 64.14a	196.50 ± 21.51b	27.50 ± 2.77d	99.20 ± 5.16c
Furfural	3.33 ± 0.63b	ND	8.46 ± 1.48a	3.21 ± 0.26b	7.69 ± 1.30c	10.31 ± 2.07b	13.25 ± 0.25a	5.71 ± 1.12d
Damascenone	1.32 ± 0.32b	0.87 ± 0.11c	1.65 ± 0.24a	1.10 ± 0.02b	0.63 ± 0.01b	0.49 ± 0.01b	1.54 ± 0.04a	ND
Nonanal	21.14 ± 0.01b	20.21 ± 0.01b	32.55 ± 0.64a	15.03 ± 0.01b	11.06 ± 0.01b	ND	17.31 ± 0.03a	ND
Total Others	79.27 ± 3.75b	56.41 ± 2.33b	101.76 ± 6.38a	55.49 ± 2.52b	305.74 ± 0.05a	229.73 ± 2.17b	74.27 ± 0.21c	133.08 ± 1.23a
Total	5084.29 ± 101.02b	4685.44 ± 62.89bc	6849.37 ± 125.33a	4438.68 ± 77.64c	5914.46 ± 20.63c	7958.70 ± 155.74b	10482.51 ± 86.57a	4000.70 ± 43.01d

Note: Mean values (SD, *n* = 3) of the same compounds followed by different letters are significantly different (p < 0.05). ND, not detected. R, red. W, white. B, black. CK, control.

In addition to ethyl octanoate and 2-methylbutyl acetate, the contents of almost all the esters from CK were lower than those of the sunshade net groups. Except for isoamyl acetate, ethyl hexanoate and 2-methylbutyl acetate in 2017 and ethyl acetate, ethyl butanoate and ethyl decanoate in both 2016 and 2017, wines of the black nets had the highest contents of all the other esters (Table 2). In 2016, the wines of the black net group had the highest content of total esters, followed by the CK, white net-treated, and, finally, red net-treated groups. However, in 2017, the red net-treated group possessed the highest total ester content, followed by the black net-treated, white net-treated and CK groups. The contents of total esters for the red net-treated group and CK were discrepant for these 2 years, but the results of the black net-treated group were shown to be consistent. Generally, the varieties of wine esters from the black net- and red net-treated groups were greater than those of the white net-treated and CK groups (Table 2).

In 2016, except for ethyl octanoate, most ester contents in grapes grown under different sunshade nets were higher than those of the CK group. The contents of total esters in grapes grown under sunshade nets were all higher than those of the CK group. In 2017, the contents of almost all kinds of esters, including the total esters, in grapes grown under black nets were higher than those of the other 3 groups. The varieties of esters in grapes grown under red and black sunshade nets were generally greater than those of the other 2 groups. Except for 1-undecanol, isooctanol and isoamyl alcohol, the contents of almost all the other alcohols in wines of grapes grown under sunshade nets were significantly higher than those of the CK group (Table 2). The contents of all the alcohols in wines of grapes grown under black nets were the highest in 2016. However, the trend was not very obvious in 2017, except for 1-hexanol, isooctanol and ethanol. The contents of the total alcohols in grapes grown under sunshade nets were markedly higher than those of the CK group in both 2016 and 2017, with the wines of the black net-treated group having the highest contents. The varieties of alcohols in wines of the white net-treated group were greater than those of the other 3 groups, while the alcohol variety of CK was the smallest.

In addition to the hexanoic acid content in wines in 2016, the contents of almost all the other acids in the wines of grapes grown under different sunshade nets were significantly higher than those of the CK group. Wines of grapes grown under black nets possessed the highest contents for all the other acids in 2016, but similar results were not observed in 2017, except for octanoic acid and acetic acid (Table 2). This finding is consistent with the results of the basic indexes (Table S1) and aromas in grapes (Table 1). Varieties of acids in wines of grapes grown under black and red nets were generally more abundant than those of the other 2 groups. Wines of the black and red net-treated groups had the highest total acidity in 2016 and 2017, respectively. For the other volatile substances, the contents of furfural, damascenone and nonanal in wines of grapes grown under different nets were generally higher than those of the CK group in both 2016 and 2017, while the wines of the black net-treated group had the highest contents. This result is consistent with results of the study by Diesler et al. (2019), who found that UV-C treatment could decrease the β-damascenone and linalool contents in wines. The same trend was also observed for the content of styrene in wines in 2016. However, the opposite trend was observed for styrene in wines of 2017, which is the year that the content of styrene in wines of the black sunshade net-treated group was the lowest, while the CK group had the highest content. However, there was no obvious similarity in the contents of *β*-cyclocitral in wines. Furthermore, wines made with grapes grown under red nets in both 2016 and 2017 had the highest content (Table 2). For the total content of volatile components, the contents in wines made with red and black net-treated grapes in 2016 were significantly higher than those of the CK group, while no obvious significant difference was observed between the white net-treated and CK groups. In 2017, aroma contents in all 3 sunshade net treated groups were notably higher than those of the CK group. Wines made with black net-treated grapes had the highest contents of aromas in both 2016 and 2017 (Table 2).

**Fig. 3:**
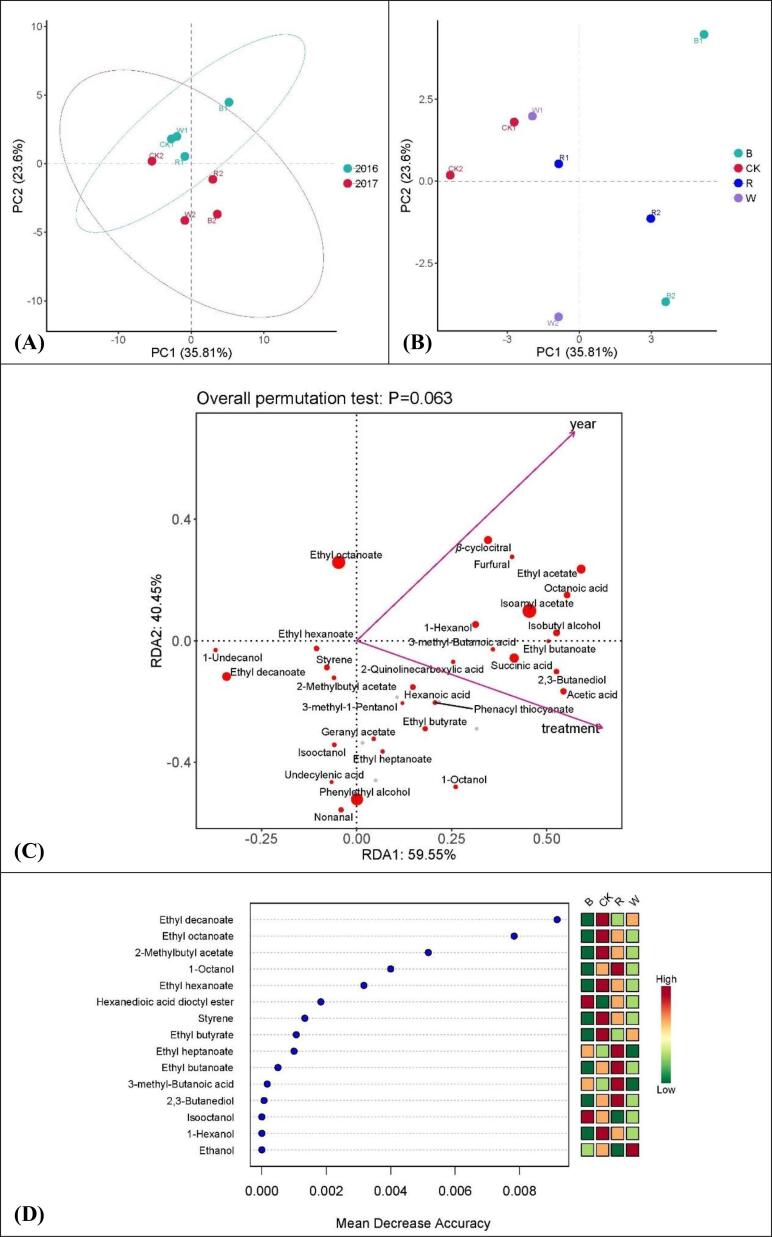
Aroma characteristics of wines. (A), PCA analysis for the effect of vintage on aroma substances. (B), PCA analysis for the effect of treatment on aroma substances. (C), RDA/CCA analysis for the characteristic aroma substances. (D), Random forest analysis for the characteristic aroma substances. R, red. W, white. B, black. CK, control group. (For interpretation of the references to color in this figure legend, the reader is referred to the web version of this article.)

In PCA analysis, PC1 and PC2 explained 35.81 % and 23.60 % of the total variance, respectively (Fig. 3A and B). In terms of vintage, wine samples were clearly distinguished by PC2, while PC1 showed low discrimination to samples (Fig. 3A). As far as treatment is concerned, B1, B2 and R2 were distinctly distinguished from the other groups by PC1, while W2, B2 and R2 were clearly distinguished from the other groups by PC2 (Fig. 3B). According to the RDA/CCA analysis results, high degree of correlation was observed for aroma substances contents with both vintage and treatment methods. It was also found that there is a positive correlation between the vintage and treatment method. Ethyl octanoate, *β*-cyclocitral, Furfural, Ethyl acetate, Octanoic acid, Styrene, 2-Methylbutyl acetate, Geranyl acetate, Isooctanol, Ethyl heptanoate, Undecylenic acid, Phenylethyl alcohol and Nonanal were influenced more by the vintage, while the other substances were more affected by the treatment methods. The contents of Ethyl octanoate, Isoamyl acetate, Phenylethyl alcohol, Ethyl decanoate, Succinic acid, Ethyl acetate and *β*-cyclocitral were apparently higher than that of the other substances. Ethyl acetate, Isoamyl acetate, Ethyl octanoate and Ethyl decanoate contributed most in ester aroma components. Octanoic acid, Succinic acid, Acetic acid and Hexanoic acid contributed most in acid aroma components. Isobutyl alcohol, 1-Hexanol, 2,3-Butanediol and Phenylethyl alcohol contributed most in alcohol aroma components (Fig. 3C). The contribution of Ethyl decanoate to the grape aromas was significantly higher than that of other substances, followed by Ethyl octanoate, 2-Methylbutyl acetate, 1-Octanol, Ethyl hexanoate and Hexanedioic acid dioctyl ester. Other substances contributed relatively small to the grape aromas (Fig. 3D).

In general, the contents of most of the aromatic substances in wines made of grapes grown under light-selective sunshade nets were higher than those of the CK group. Wines made from the net treatment group also have more varieties of aromas. The black net-treated group usually had the greatest variety and highest content of aromatic substances, indicating that the light-selective sunshade net is a potential ideal cultivation measure in vineyards to improve the varieties and contents of aromatic substances in wines.

### 3.5 Odor profiles

#### 3.5.1 Odor profiles of grapes

According to the odor properties of different aromatic substances detected in grapes, we summarized the aromatic contents of different series in grapes under different treatment conditions (Fig. S3). The black net-treated group had the highest fruity content in 2016, followed by the red net-treated, CK and white net-treated groups. In 2017, by contrast, the contents of fruity aromas of all 3 net-treated groups were significantly higher than those of the CK group, while the fruity aroma content of the black net-treated group was much higher, almost 120–170 μg/L more than that of the other 3 groups. The contents of floral aromas were very low in both 2016 and 2017, but significant differences were still observed. The black net-treated group had the highest content in 2016, followed by the white net-treated group, while there was no obvious difference between the red net-treated and CK groups. In 2017, the contents of fruity aromas of all 3 net-treated groups were significantly higher than those of the CK group, with the sequences being black net-treated, red net-treated, white net-treated and CK. The black net-treated group had the highest content of sweet aromas in 2016, followed by the white net-treated, red net-treated and CK groups. In 2017, the black net-treated group still possessed the highest content, but the content of CK was higher than that of white net- and red net-treated groups. The sequence for the green aroma content in 2016 was black net-treated, red net-treated, CK and white net-treated. However, in 2017, the sequence was black net-treated, CK, red net-treated and white net-treated. For chemical aromas, the black net-treated group had the highest contents for both 2016 and 2017, while no significant differences were observed for the other 3 groups. The citrus aroma content of the red net-treated group was the highest in 2016, while that of the white net-treated group was the lowest. No obvious difference was found between the red net-treated and black net-treated groups. However, in 2017, the CK group had the highest content, followed by the red net-treated, black net-treated and CK groups. In general, fruity aromas were the most abundant in both 2016 and 2017, followed by green, sweet, citrus and chemical aromas. The floral aroma was the least abundant, with very low detected amounts. Generally, compared with the CK group, the light-selective sunshade net treatment, especially the black net and red net treatments, was effective in improving the fruity, floral and sweet contents of grapes. This treatment was good for improving the quality of grapes. However, the contents of unwelcome green and chemical aromas were also relatively high for the black net-treated groups. This result was a disadvantage. White nets could decrease the green and citrus aromas, which were thought to have a negative influence on the comprehensive quality of grapes during harvest (Schelezki, Šuklje, Boss, & Jefferya, 2018). Further studies need to be conducted to estimate wine qualities so that the effect of light-selective sunshade net treatment can be synthetically evaluated.

#### 3.5.2 Odor profiles of wines

The odor profile is a good tool to evaluate the taste and quality of wine (Ferrero-del-Teso et al., 2020). Although numerous aromatic substances were detected in the wines of different groups, not all of the components could influence the aromatic characteristics of wines to a remarkable degree. In general, it is widely believed that only the components whose odor activity values (OAVs) are above 1 can significantly affect the taste properties of the corresponding products (Wen et al., 2014). To evaluate the contribution of diverse aromatic substances to the mouthfeel and flavor of wines, odor activity values (OAVs) were calculated by dividing the concentration of the detected aroma compounds by the corresponding odor thresholds (OTs), which were obtained from the literature. Compounds whose OAVs were above 1 were selected, and the corresponding odor properties were described. Table 3 shows the OT, odor descriptions, aromatic series and the relative OAVs of most potent volatiles in wines. Obviously, the OAVs of isoamyl acetate, ethyl octanoate, damascenone, *β*-cyclocitral and nonanal were higher than 20, indicating that these compounds may greatly influence wine aromas.

**Table 3:** Odour thresholds (OT), odour description, odour activity values (OAVs) of most potent volatiles in wines.

Compounds	OT	Odour description	Aromatic series	Odour activity value							
				2016				2017			
				R	W	B	CK	R	W	B	CK
Ethyl butyrate	20	Strawberry	Fruity	0.42	1.00	1.09	0.47	0.68	0.47	0.88	0.52
Isoamyl acetate	30	Banana, pear, apple, sweet	Fruity, sweet	11.80	10.93	15.66	11.52	21.97	14.55	16.97	10.81
Ethyl octanoate	2	Fruity, cream, milk, sweet	Fruity, sweet	164.10	151.57	210.94	185.55	193.49	131.03	245.74	225.18
Ethyl hexanoate	14	Green apple, strawberry	Fruity	0.95	1.45	1.53	1.49	1.59	1.23	1.15	1.25
Ethyl decanoate	100	Sweet, grape, fatty	Sweet, fruity, fatty	0.36	1.89	1.13	2.03	0.78	1.24	0.66	0.84
Geranyl acetate	9	Floral	Floral	0.86	0	1.10	0.59	0.59	0.23	0.62	0
Ethyl butanoate	1	Pineapple	Fruity	1.32	0.75	0.89	0.66	1.26	1.17	1.78	0.63
2-Methylbutyl acetate	5	Banana	Fruity	0.98	0.73	1.24	1.07	1.1	0.84	0.97	0.87
1-Hexanol	30	Green, fruity, mellow, sweet	Green, fruity, sweet	1.39	1.45	1.67	1.40	1.61	1.37	2.23	1.40
Damascenone	0.05	Sweet, woody, fruity, floral, green	Sweet, fruity, floral, green	26.40	17.40	33	22	12.60	9.80	30.80	0
*β*-cyclocitral	5	Sweet, floral, herbaceous	Floral, sweet	6.20	2.24	5.36	3.29	51.77	39.30	5.50	19.84
Nonanal	1	Wax, orange, flower	Citrusy, Floral	21.14	20.21	32.55	15.03	11.06	0	17.31	0

Note: Odour Activity Values (OAVs), ratio of the concentration of a molecule to its odour threshold. OT, Odour threshold (ppb in water), all the thresholds were obtained from references (Belisario-Sánchez et al., 2011, Fariña et al., 2015, Pino and Mesa, 2006, Wen et al., 2014). R, red. W, white. B, black. CK, control.

The descriptors of a component might not be unique. One compound could be described by several different descriptors (Wen et al., 2014). Several different descriptors might be grouped into the same aroma series. For example, descriptors such as apple, banana and strawberry belong to the same aromatic series of fruity. Therefore, the OAVs of the aromatic compounds that had similar odor descriptions were grouped. Six aromatic series of odors were established, including fruity, sweet, fatty, floral, green and citrus odors (Table 3). Radar maps of aromatic series were then obtained for the wines under different treatment conditions in 2016 and 2017 and are shown in Fig. S4. In this figure, only components with an OAV above 1 were chosen. The coordinates for each series were the summations of the corresponding OAVs values. As shown in Fig. S4, fruity was the most intense odor of the wines in 2016, followed by sweet and floral. The OTs of ethyl octanoate and damascenone were low. The fruity odor mainly comes from ethyl octanoate, damascenone and isoamyl acetate. The sweet odor was chiefly from isoamyl acetate, ethyl octanoate, damascenone and *β*-cyclocitral. The floral odor is mainly due to damascenone and *β*-cyclocitral. The intensities of green and citrusy aromas were low, chiefly from damascenone and nonanal, with low OTs, respectively. The intensity of fatty acids was extremely low and mainly from ethyl decanoate. From Fig. S4, we could conclude that the odor characteristic structures of wines from grapes receiving different treatments in 2016 were similar. However, the intensities of the main odor properties of the wine from the black net group were significantly higher than those of the other three groups. There was no large difference in the odor properties of the wines from the red net-treated, white net-treated and CK groups, while the intensities of the fruity odor of CK and the floral odor of the red net-treated group were slightly higher than those of the other 2 groups. The trends of odor characteristics of the wines in 2017 were observed to be similar to those in 2016 (Fig. S4). The floral odor of CK was obviously lower than that of the other 3 groups, while the floral intensity of the red net-treated group was the highest. However, the intensities of the sweet and fruity odors were extraordinarily lower than those of the other groups. The aroma qualities of wines from CK and white net-treated groups were inconsistent.

## 4 Conclusion

Light-selective sunshade nets were proven to significantly decrease the radiation intensity. The treatments decreased the sugar contents in both grapes and wines, meanwhile, increased the acid contents. This is beneficial for improving wine grape quality in northwest China regions such as Ningxia. The contents of TP, TT and TFA in grapes were increased, while the TFD and TA contents were decreased. The contents of the majority of phenolic substances in wine were increased. Most of the aromatic substance contents in grapes and wines receiving light-selective sunshade net treatment were higher than those of the control group. Red and black nets could improve pleasant aromas, such as fruity, floral and sweet aromas, while white nets could decrease pleasant flavors. Overall, light-selective sunshade nets, especially the black net, were shown to be an ideal material for solving the problem of relatively high sugar and low acid contents in Northwest China. Simultaneously, light-selective sunshade nets were proven to be beneficial to the development of most phenolic substances and aromas. However, each kind of net has its own advantages and disadvantages. Which kind of net should we use? Should we combine several nets with different colors? The situation will depend on the specific circumstances in different regions. In addition, light-selective sunshade nets can not only decrease the solar radiation intensity but also influence the temperature and humidity. Thus, the relative changes may be the result of multifactor interactions. Which factor plays the main role? What should we do in production practice? Further efforts are needed to interpret the influencing mechanism in more detail.

## CRediT authorship contribution statement

**Yanlun Ju:** Resources. **Uwamahoro Francoise:** Conceptualization, Methodology, Software, Investigation, Writing – original draft. **Demei Li:** . **Yang Zhang:** Conceptualization, Methodology, Software, Investigation, Writing – original draft. **Bochen Liu:** Conceptualization, Methodology, Software, Investigation, Writing – original draft. **Miao Sun:** . **Yulin Fang:** Resources. **Xiaofeng Wei:** Conceptualization, Methodology, Software, Investigation, Writing – original draft.

## Declaration of Competing Interest

The authors declare that they have no known competing financial interests or personal relationships that could have appeared to influence the work reported in this paper.
