# Supplementary material for: Effect of light-selective sunshade net on the quality and aromatic characteristics of Cabernet Sauvignon grapes and wine: Exploratory experiment on strong solar irradiance in northwestern China
Source: Food Chem X. 2022 Nov 14;17:100510. doi: 10.1016/j.fochx.2022.100510 (PMC9943764; doi:10.1016/j.fochx.2022.100510)
Supplement: Supplementary data 1 [file mmc1.doc]

**Supplementary materials**

**Fig. S1**. Field treatment diagram. R, red net. W, white net. B, black net. CK, control group.

**Fig. S2**. Solar radiation intensities under different treatments. R, red. W, white. B, black. CK, control group. Different letters indicate significantly difference values (p < 0.05).

**Fig. S3**. Aromas contents of different series in grapes under different treatments. R, red. W, white. B, black. CK, control group. Different letters indicate significantly difference values (p < 0.05).

**Fig. S4**. Radar map of wine aroma characteristics. R, red. W, white. B, black. CK, control group.


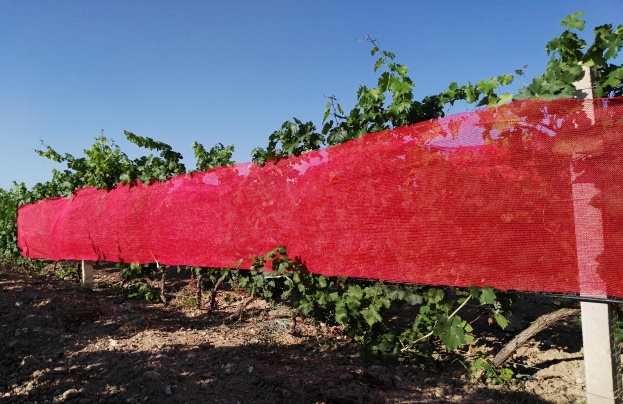

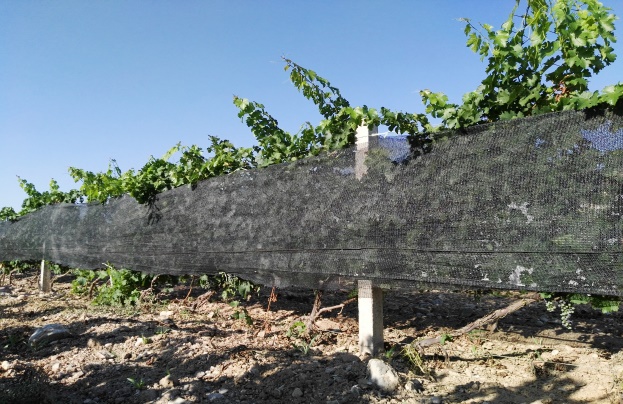

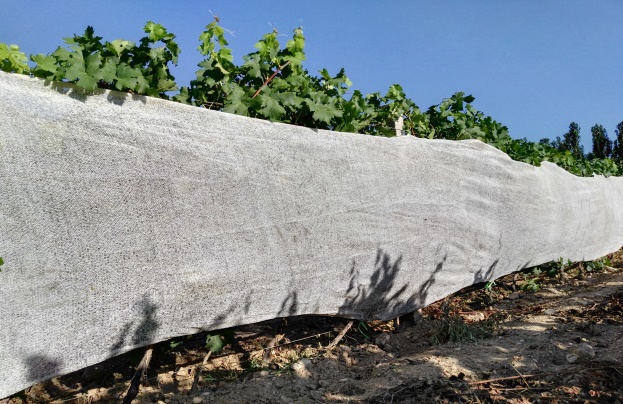

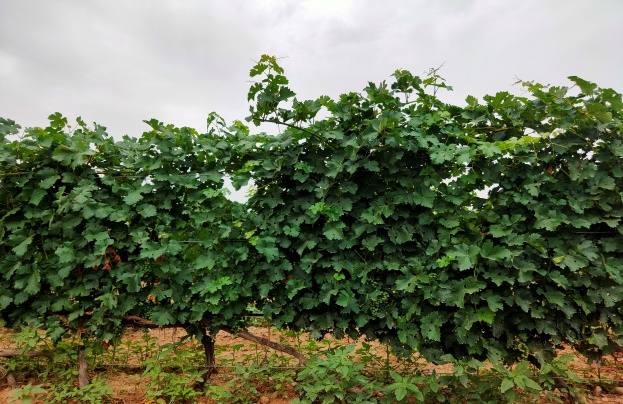


**Fig. S1.**


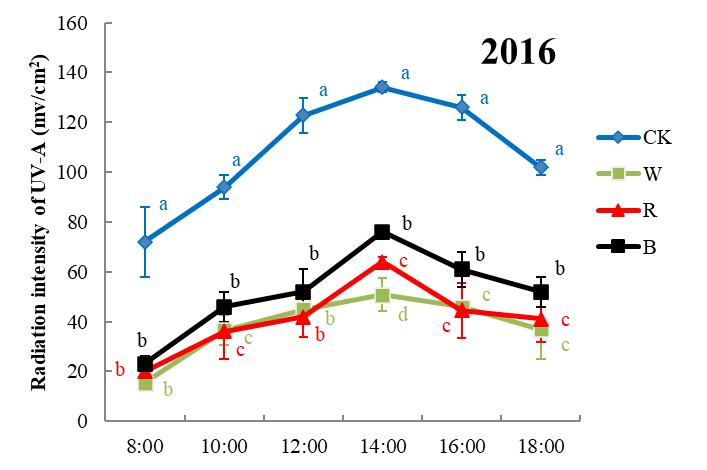

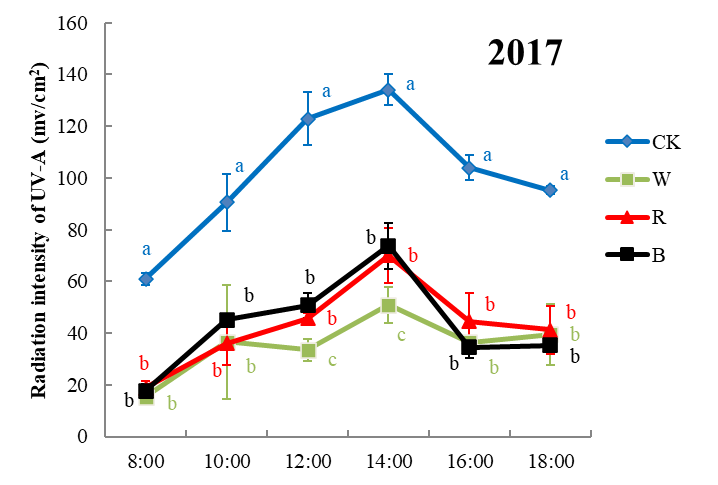


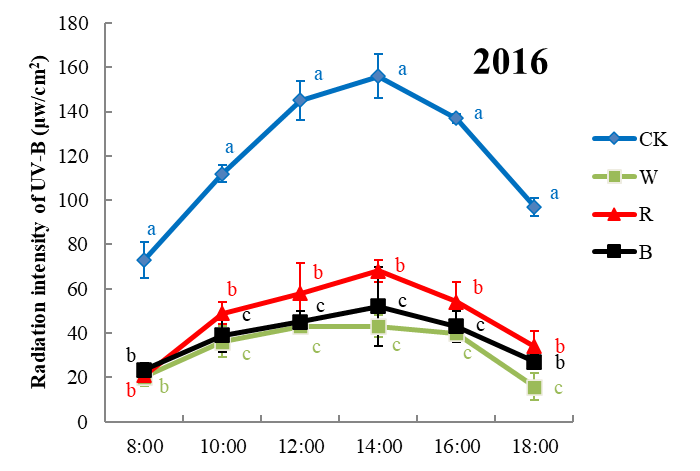

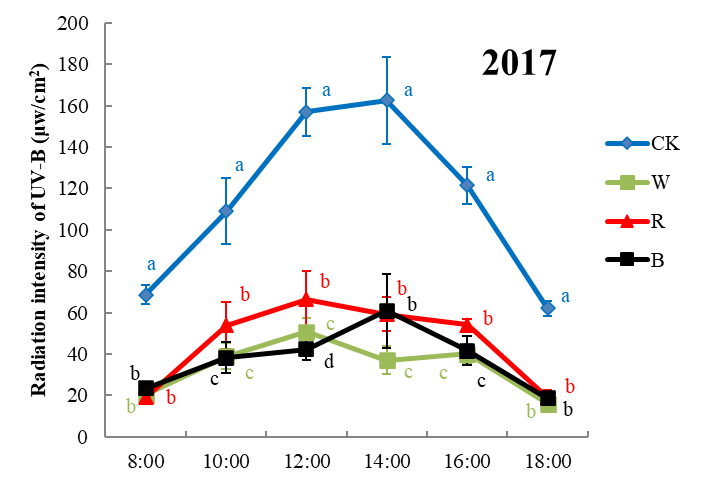


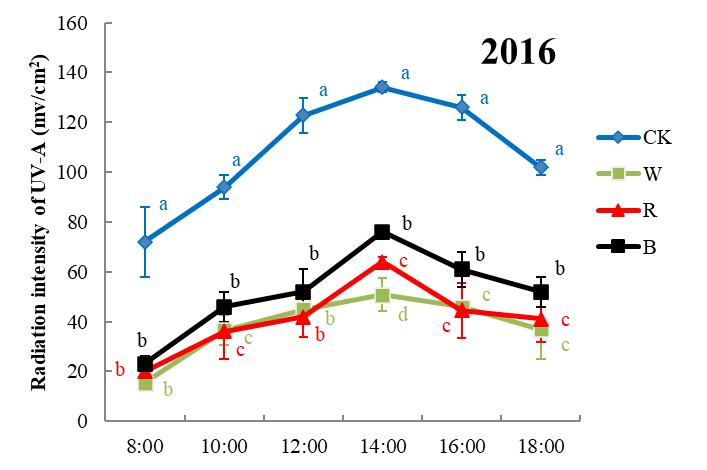

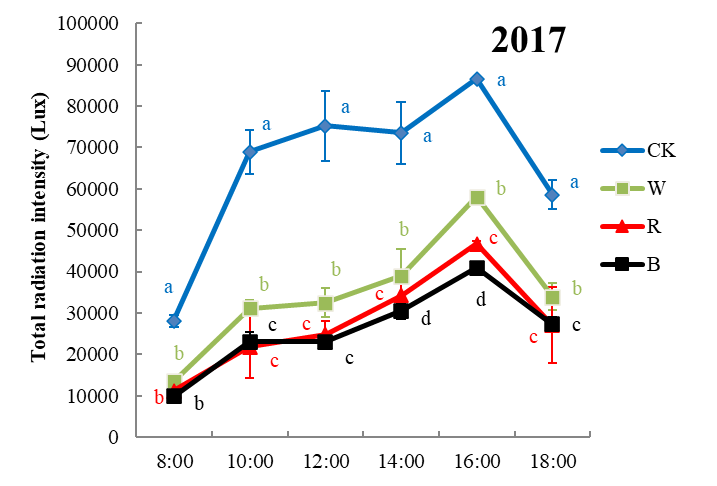


**Fig. S2.**


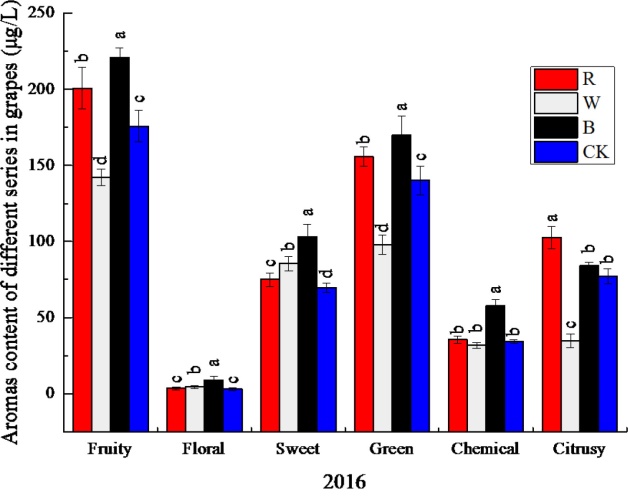

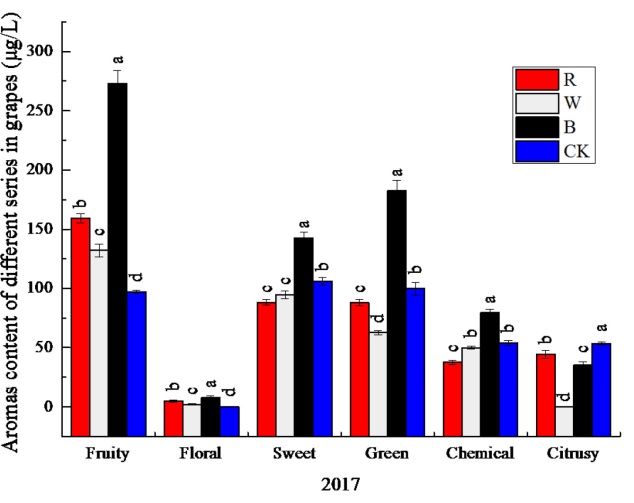
**Fig. S3.**


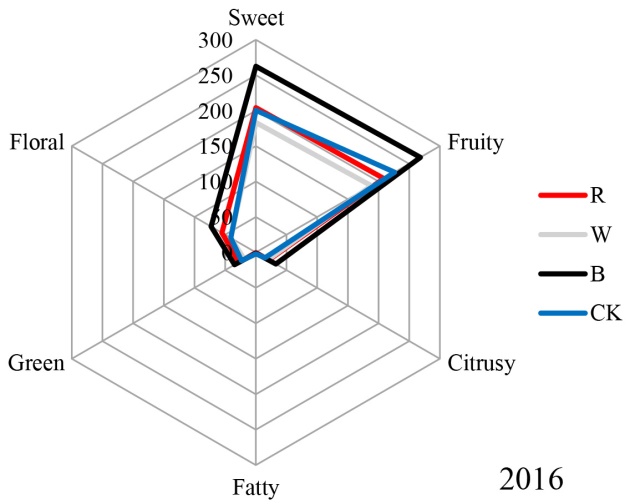

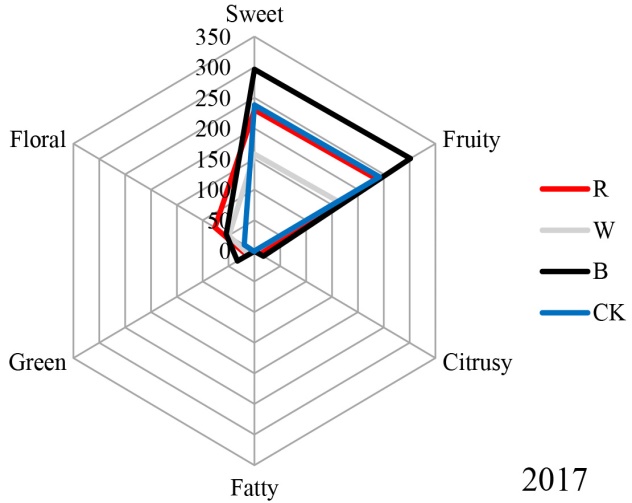


**Fig. S4.**

**Table S1 Basic physiological indexes under the light-elective sunshade nets**

| Indexes | 2016 | | | | 2017 | | | |
| --- | --- | --- | --- | --- | --- | --- | --- | --- |
|  | R | W | B | CK | R | W | B | CK |
| *Grapes* | | | | | | | | |
| Transverse diameter/cm | 1.26 ± 0.12a | 1.24 ± 0.11a | 1.14 ± 0.08b | 1.13 ± 0.11b | 1.12 ± 0.18a | 1.15 ± 0.14a | 1.16 ± 0.18a | 1.01 ± 0.09b |
| Vertical diameter/cm | 1.31 ± 0.22a | 1.28 ± 0.21a | 1.17 ± 0.13b | 1.16 ± 0.5b | 1.14 ± 0.13b | 1.20 ± 0.11a | 1.20 ± 0.24a | 1.13 ± 0.35b |
| °Brix | 22.72 ± 1.21c | 23.06 ± 0.04b | 20.67 ± 1.13d | 25.32 ± 0.17a | 24.92 ± 0.07c | 25.20 ± 1.15b | 22.79 ± 0.09d | 27.40 ± 1.12a |
| pH | 3.46 ± 0.16b | 3.53 ± 0.33b | 3.16 ± 0.25c | 3.72 ± 0.11a | 3.32 ± 0.16bc | 3.66 ± 0.18ab | 3.15 ± 0.10c | 3.93 ± 0.23a |
| Total acidity (g·L^-1^) | 4.34 ± 0.16a | 4.22 ± 0.39a | 4.87 ± 0.47a | 3.62 ± 0.54b | 4.85 ± 0.01b | 4.47 ± 0.08c | 5.14 ± 0.02a | 3.54 ± 0.06d |
| *Wines* | | | | | | | | |
| Total acidity (g·L^-1^) | 7.90 ± 1.17b | 6.31 ± 0.26c | 9.36 ± 1.30a | 5.11 ± 0.52d | 7.27 ± 0.07 b | 6.58 ± 1.10 b | 8.96 ± 0.45a | 5.32 ± 0.31c |
| Total sugar (g·L^-1^) | 4.56 ± 0.35b | 5.12 ± 0.64b | 3.42 ± 0.24c | 6.32 ± 1.07a | 3.94 ± 0.10bc | 4.41 ± 0.84b | 3.27 ± 0.33c | 5.73 ± 0.46a |
| Alcohol content (v/v) | 12.54 ± 0.01%c | 13.15 ± 0.01%b | 12.01 ± 0.00%d | 14.21 ± 0.02%a | 12.76 ± 0.01%b | 12.79 ± 0.02%b | 12.25 ± 0.03%c | 13.97 ± 0.00%a |

**Note:** Mean values (SD, *n* = 3) of the same compounds followed by different letters are significantly different (p < 0.05). R, red. W, white. B, black. CK, control. Different letters indicate significantly difference values (p < 0.05).
